# Structural modification of functionalized carbazoles: from *Z′* = 1 to *Z′* = 6

**DOI:** 10.1107/S2052520626004506

**Published:** 2026-05-19

**Authors:** Matokah Abualnaja, Michael Richard Probert, Michael John Hall, Carolyn P. Brock, Paul Gordon Waddell

**Affiliations:** a Chemistry, School of Natural and Environmental Sciences Newcastle University Newcastle upon Tyne NE1 7RU United Kingdom; b Department of Chemistry Umm Al-Qura University Mecca Saudi Arabia; c Department of Chemistry University of Kentucky Lexington Kentucky40506 USA; Academy of Sciences of the Czech Republic, Czechia

**Keywords:** high *Z*′, carbazole, heterocycle, crystal packing

## Abstract

Crystal structures with asymmetric units comprising large numbers of molecules (*Z*′ > 4) remain rare. Their study provides a unique opportunity to further the understanding of the physical basis for crystal formation, supporting the advancement of crystal structure prediction, crystal engineering and the study of polymorphism. Here the crystal structures of a series of three related 2,3,9,9*a*-tetra­hydro-1*H*-carbazoles are discussed, one of which exhibits an asymmetric unit comprising six crystallographically independent molecules (*Z*′ = 6). Careful analysis of this structure reveals approximate *Fdd*2 symmetry and strong evidence for this being linked to a symmetry-reducing, low-temperature phase transition. Comparison of the structures reveals that the variation in *Z*′ values is linked to conformational changes related to the lack of classical hydrogen bonding in the high-*Z*′ structure.

## Abstract 2

The crystal structure of a 2,3,9,9*a*-tetra­hydro-1*H*-carbazole in the space group *Ia* is reported, showing *Z*′ = 6 and approximate *Fdd*2 symmetry, and compared to the low *Z*′ crystal structures of two closely related congeners.

## 1. Introduction

**Figure 1:**
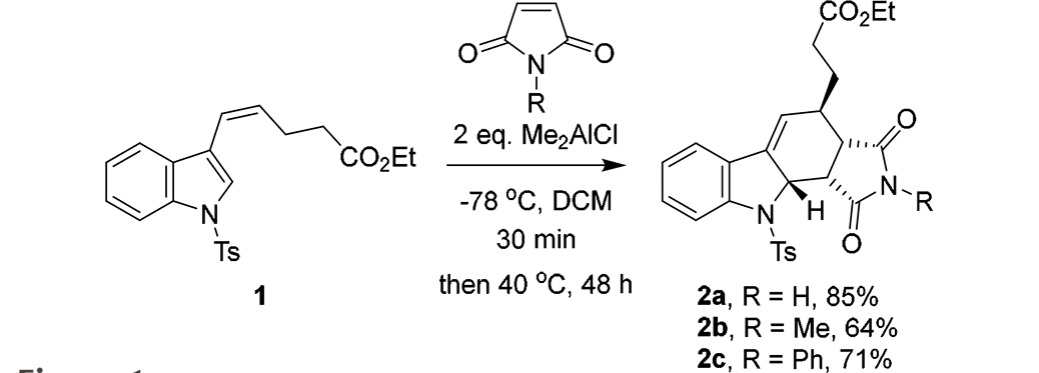
Synthesis of racemic 2,3,9,9*a*-tetra­hydro-1*H*-carbazoles (**2a**–**2c**).

The carbazole scaffold is a common motif in natural product and medicinal chemistry (Schmidt *et al.*, 2012 ▸), being found in a wide range of bioactive alkaloids such as the anti-cancer staurosporines (Nakano & Ōmura, 2009 ▸) and their semi-synthetic analogues, such as the kinase inhibitor midostaurin (Stone *et al.*, 2018 ▸). As part of our work towards the synthesis of highly functionalized carbazoles, we have previously reported the preparation and subsequent functionalization of a wide range of 2,3,9,9*a*-tetra­hydro-1*H*-carbazoles, themselves formed via Diels-Alder reactions of 3-alkenyl-1*H*-indoles (Cowell, Abualnaja *et al.*, 2015 ▸; Cowell, Harrington *et al.*, 2015 ▸; Abualnaja *et al.*, 2016 ▸; Abualnaja *et al.*, 2021 ▸). As reported previously (Cowell, Abualnaja *et al.*, 2015 ▸), 2,3,9,9*a*-tetra­hydro-1*H*-carbazoles (**2a**–**2c**) were synthesized as single diastereomers via a Lewis acid catalysed Diels–Alder reaction between ethyl (*Z*)-5-(1-tosyl-1*H*-indol-3-yl)pent-4-enoate (**1**) and 1*H*-pyrrole-2,5-dione, 1-methyl-1*H*-pyrrole-2,5-dione or 1-phenyl-1*H*-pyrrole-2,5-dione, respectively (Fig. 1 ▸). The crystal structure of **2a** was first reported in that article.

As a part of the wider research project that produced the structure of **2a**, the structures of **2b** and **2c** were also determined. Where the structure of **2c** is largely unremarkable, that of **2b** crystallized with a surprisingly large number of crystallographically independent molecules in the asymmetric unit. The number of molecules in the asymmetric unit of a homomolecular structure is denoted by the value *Z*′ and in the case of **2b**, *Z*′ is particularly high, with a value of 6. High-*Z*′ structures are rare. Those with *Z*′ ≥ 6 represent about 0.1% of the over 1.4 million structures in the CSD (v. 6.01, Nov. 2025) and, in general, the greater the *Z*′ value, the less frequently they are observed (Steed & Steed, 2015 ▸).

In crystal structures with high-*Z*′ values the molecules in the asymmetric unit are often related by approximate, and not crystallographic, symmetry (Brock, 2016 ▸; Waddell, 2025 ▸). Such approximate symmetry was apparent in the structure of **2b** and therefore the packing in this structure was investigated carefully.

The structure of **2b**, which was determined in the space group *Ia* at 150 K, was found to exhibit approximate *Fdd*2 symmetry to such a degree that it is likely that the structure is truly *Fdd*2 at room temperature. Comparisons to the structures of **2a** and **2c** demonstrate that hydrogen bonding, which is dependent on systematic modifications of a single peripheral imide functional group, plays a very important role in determining the packing of this family of structures.

The need to understand, rationalize and ultimate predict the packing behaviour of molecules in the solid-state stems from both purely academic interest and as well as more practical concerns such as the design of solid-state materials and for predicting polymorphism, notably in active pharmaceutical ingredients (Datta & Grant, 2004 ▸). Through the study of these structures, we were presented with the opportunity to analyse these structures in order to rationalize these values. These results could then be expected to inform the fields of crystal engineering and crystal structure prediction for which anticipating high-*Z*′ structures still proves to be very difficult (Waddell, 2025 ▸).

## 2. Materials and methods

### 2.1. Synthesis and single-crystal growth of compounds **2a**–**2c**

Compounds **2a**–**2c** were synthesized based on the following general method. Di­methyl­aluminium chloride (1.0 *M* in hexane or thf, 2 eq.) was added dropwise to a solution of 1*H*-pyrrole-2,5-dione, 1-methyl-1*H*-pyrrole-2,5-dione or 1-phenyl-1*H*-pyrrole-2,5-dione as appropriate (1 eq.) in dry dichloromethane (DCM) (3 ml mmol^−1^) at 195 K. The reaction mixture was stirred for 30 min, following which a solution of ethyl-(*Z*)-5-(1-tosyl-1*H*-indol-3-yl)pent-4-enoate (**1**) (1 eq.) in dry DCM (3 ml mmol^−1^) was added dropwise at 195 K. The reaction mixture was then warmed to reflux for 48 h, quenched with saturated NaHCO_3_ (aq.) (3 ml mmol^−1^) and extracted with DCM, washed with brine and dried over MgSO_4_. The solvent was removed under reduced pressure and the resulting material purified by column chromatography to give racemic 2,3,9,9*a*-tetra­hydro-1*H*-carbazoles **2a** (64%), **2b** (85%) and **2c** (71%) (Cowell, Abualnaja *et al.*, 2015 ▸). Crystals suitable for crystallographic analysis were grown *via* slow evaporation of the solvent from a solution of the compound in di­chloro­methane (**2a** and **2c**) or chloro­form (**2b**). Analysis of these compounds (^1^H and ^13^C NMR spectra) are available in the electronic supporting information of the original article (Cowell, Abualnaja *et al.*, 2015 ▸).

### 2.2. Single-crystal X-ray diffraction

Single-crystal diffraction data for all samples were collected at 150 K on an Xcalibur, Atlas, Gemini Ultra diffractometer equipped with an Oxford Cryosystems CryostreamPlus open-flow N_2_ cooling device. Data for **2b** were collected using copper X-ray radiation [λ(Cu *K*α) = 1.54184 Å] and the intensities were corrected for absorption empirically using spherical harmonics. Data for **2c** were collected using molybdenum X-ray radiation [λ(Mo *K*α) = 0.71073 Å] with the intensities being corrected for absorption using a multifaceted crystal model created by indexing the faces of the crystal for which data were collected (Clark & Reid, 1995 ▸). Cell refinement, data collection and data reduction were undertaken via the software *CrysAlisPro* (Rigaku Oxford Diffraction, 2014 ▸).

All structures were solved using *XT* (Sheldrick, 2015 ▸) and refined by *XL* (Sheldrick, 2008 ▸) using the *Olex2* interface (Dolomanov *et al.*, 2009 ▸). All non-hydrogen atoms were refined anisotropically and hydrogen atoms were positioned with idealized geometry, with the exception of those bound to heteroatoms, the positions of which were located using peaks in the Fourier difference map. The displacement parameters of the hydrogen atoms were constrained using a riding model with *U*_(H)_ set to be an appropriate multiple of the *U*_eq_ value of the parent atom.

The structure of **2a** has been reported previously (Cowell, Abualnaja *et al.*, 2015 ▸) (CSD refcode: QOVQID; CCDC number: 2524699). The structures of **2b** and **2c** have CCDC numbers 2043589 and 2043590, respectively.

## 3. Analysis of the approximate symmetry in **2b**

**Figure 2:**
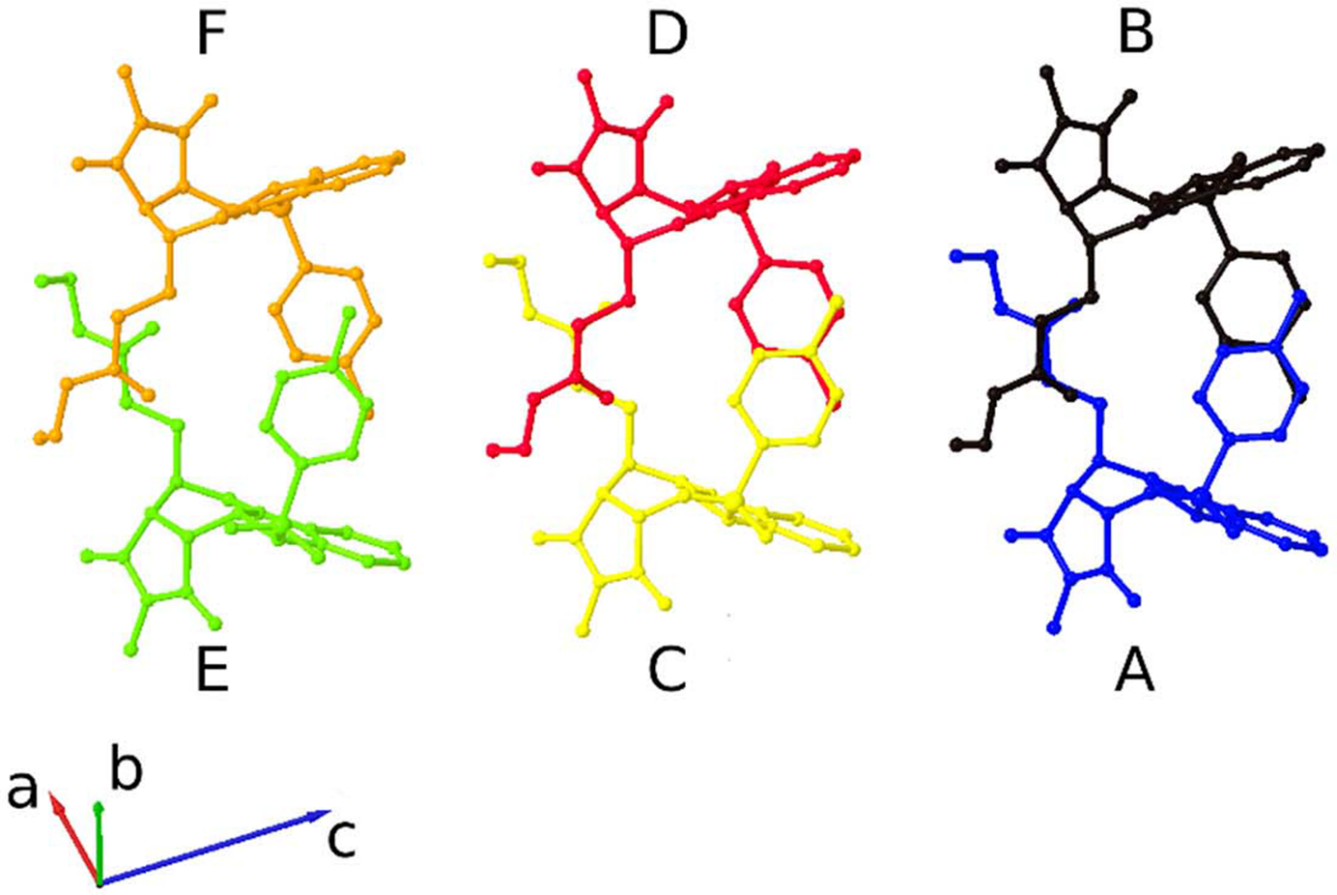
The asymmetric unit of **2b**. The crystallographically independent molecules of the asymmetric unit are labelled A–F.

**Figure 3:**
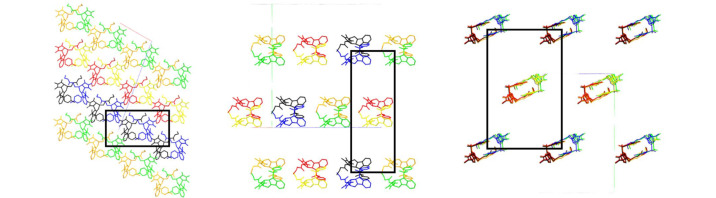
The structure of **2b** viewed along the [010] (left), [101] (centre) and [105] (right) directions highlighting the centring on each face of the approximate *Fdd*2 cell.

An approximate twofold rotation around [101] in structure **2b** and an approximate translation [101]/3 are obvious (Fig. 2 ▸). Direction [101] is perpendicular to the reflection planes of the space group glides *a* and *c*, so it seemed possible that a smaller, basic unit cell could have point symmetry *mm*2. An approximate *Fdd*2 cell was then found; the *F* centring is shown in Fig. 3 ▸. The *Fdd*2 cell can be related to the *Ia* cell by the transformation: ***a****_Fdd_*_2_ = (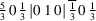)***a****_Ia_* The determinant of that matrix is ⅔ so that *Z_Fdd_*_2_ = (⅔)(4)(6) = 16 and *Z*′_*Fdd*__2_ = 1. The angle β_*Fdd*2_ is 89.98°. The ratio of the intensities of reflections that would have integral indices in the *Fdd*2 cell to those that would have fractional indices is 5.7.

Because the compound is no longer available it is impossible to be sure that the *Ia*, *Z*′ = 6 structure found at 150 K would transform to an *Fdd*2, *Z*′ = 1 structure at some higher temperature. While the proposed transition can only be a hypothesis, the evidence for such a transition is very strong.

(1) The *Continuous Symmetry Measure* calculated for *C*2 symmetry using the *CSM* software (Zabrodsky *et al.*, 1992 ▸; Alon & Tuvi-Arad, 2018 ▸) is only 0.014. Values for pairs of molecules A-B, C-D, and E-F are 0.023, 0.012, and 0.066.

(2) The approximate translation [101]/3 is very exact. Analysis of the approximate translation using the software of Brock & Taylor (Brock & Taylor, 2020 ▸) gave an rmsd of 0.40 Å, which is small, with fractional conformational, orientational, transverse, and longitudinal components accounting for 37%, 20%, 37%, and 6% of the total.

The conformations of the six molecules are very similar. The 15 rmsds calculated for molecular overlay with the CCDC program *Mercury* (Macrae *et al.*, 2020 ▸) average 0.14 Å; the range is 0.05–0.24 Å.

The centroids of molecule pairs are evenly spaced along [101]. Translations between centroids of adjacent pairs A-B, C-D, E-F differ from [⅓ 0 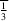] by [−0.003, −0.003, −0.004], [−0.017, 0.003, −0.004], and [0.021, 0.000, 0.007].

The transverse component of the modulation is very small. The centroids for pairs A-B, C-D, and E-F are offset from the approximate twofold axis by 0.14, 0.09, and 0.19 Å. The small CSM values show that the molecular orientations are very similar.

(3) The *Ia* structure is twinned. Twinning often accompanies a phase transition to a lower-symmetry structure during which the crystal is said to have remained single, which is to say that it continues to diffract well even after it becomes twinned.

The twin law found for the *Ia* structure is consistent with a transition from an *Fdd*2 structure. For a full description of the twinning see the supplementary information.

(4) Refinement with *Shelxl* of the approximate *Fdd*2 structure using only the 3812 reflections that have integral indices after the transformation and that are independent after *mmm* averaging was successful (CCDC 2524699). The *R*1 value for anisotropic refinement (325 variables) and including H atoms at calculated positions is 0.069 for reflection with *I* > 2*σ*(*I*). The atomic ellipsoids are only a little suspicious. All contacts shorter than the sum of the van der Waals radii are also found for at least two molecules in the *Z*′ = 6 structure.

Additional information about the analysis of the approximate symmetry is available with the supplementary material.

## 4. Results and discussion

**Figure 4:**
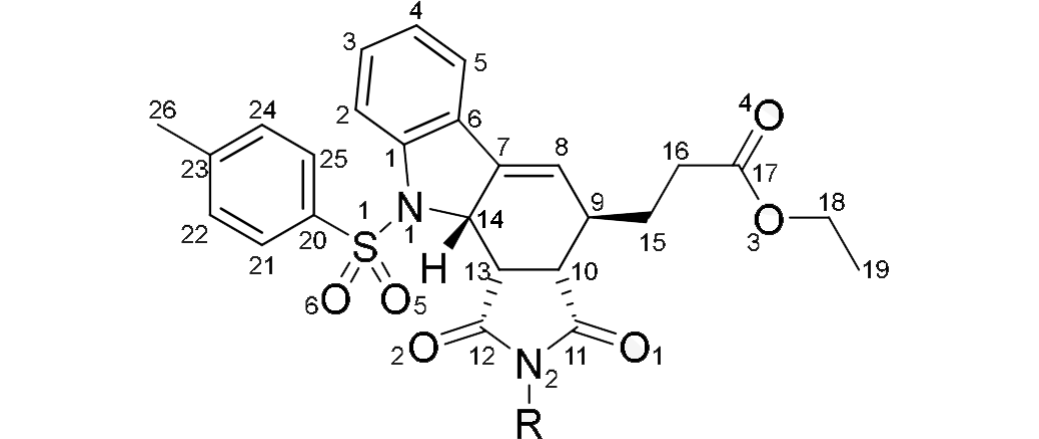
Molecular fragment common to **2a**–**2c** with atomic numbering [*R* = H (**2a**), Me (**2b**) or Ph (**2c**)].

The molecules comprising the three structures differ only in the substituent at the imide nitro­gen [*R* = H (**2a**), Me (**2b**) or Ph (**2c**)] (Fig. 4 ▸).

Within the asymmetric unit of **2b** the six molecules can be described as three discrete dimer units (A-B, C-D and E-F) formed of interactions between the faces of the carbazole and edges of the tosyl moieties such that the benzene rings of the tosyl groups appear to overlap (Fig. 2 ▸). The molecules of the dimer unit E-F differ very subtly in their relative orientation compared to those of the others leading to a pronounced translational modulation, which ultimately gives rise to the high-*Z*′ value of 6 for this structure.

**Figure 5:**
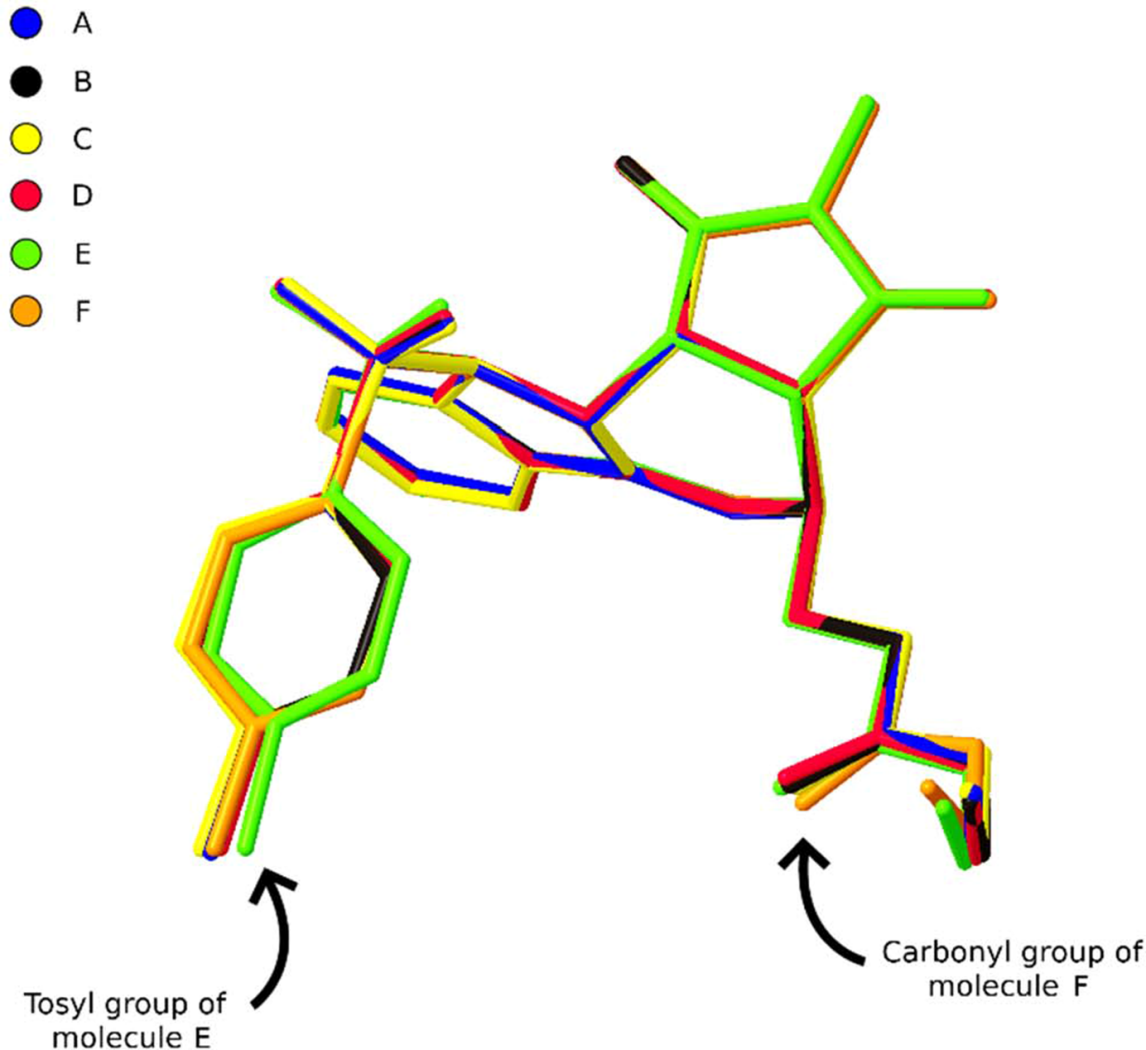
A molecular overlay diagram of the six independent molecules of **2b**. The perturbations of molecules E and F at the root of the translational modulation are highlighted.

Overlaying the six molecules in the asymmetric unit highlights that there is very little conformational variation between them (Fig. 5 ▸). What differences there are manifest in the torsion angles about the tosyl group and the ethyl ester moiety of molecules E and F, respectively, the molecules comprise the dimer unit responsible for the translational modulation.

The effect of the approximate symmetry observed in the asymmetric unit on the crystal structure as a whole allows the structure to be interpreted as a modulated form with approximate space group symmetry *Fdd*2 structure with *Z*′ = 1.

Having rationalized the high-*Z*′ value of **2b**, comparing the structure to those of the closely related congeners **2a** and **2c**, both of which have a *Z*′ of 1, may shed further light on its formation. What is immediately obvious when comparing the three structures is that **2b** is the outlier in that it is does not form classical hydrogen bonds. Where **2a** forms hydrogen bonds to molecules of itself, **2c** incorporates water molecules, most likely from being crystallized in air, to form a hydrogen bonding network comprising these water molecules and molecules of **2c**. Interactions of this kind are conspicuously absent in the structure of **2b**; there are no classical hydrogen bonds but pairs of molecules form the compact dimers with approximate twofold rotation symmetry observed in the asymmetric unit.

**Figure 6:**
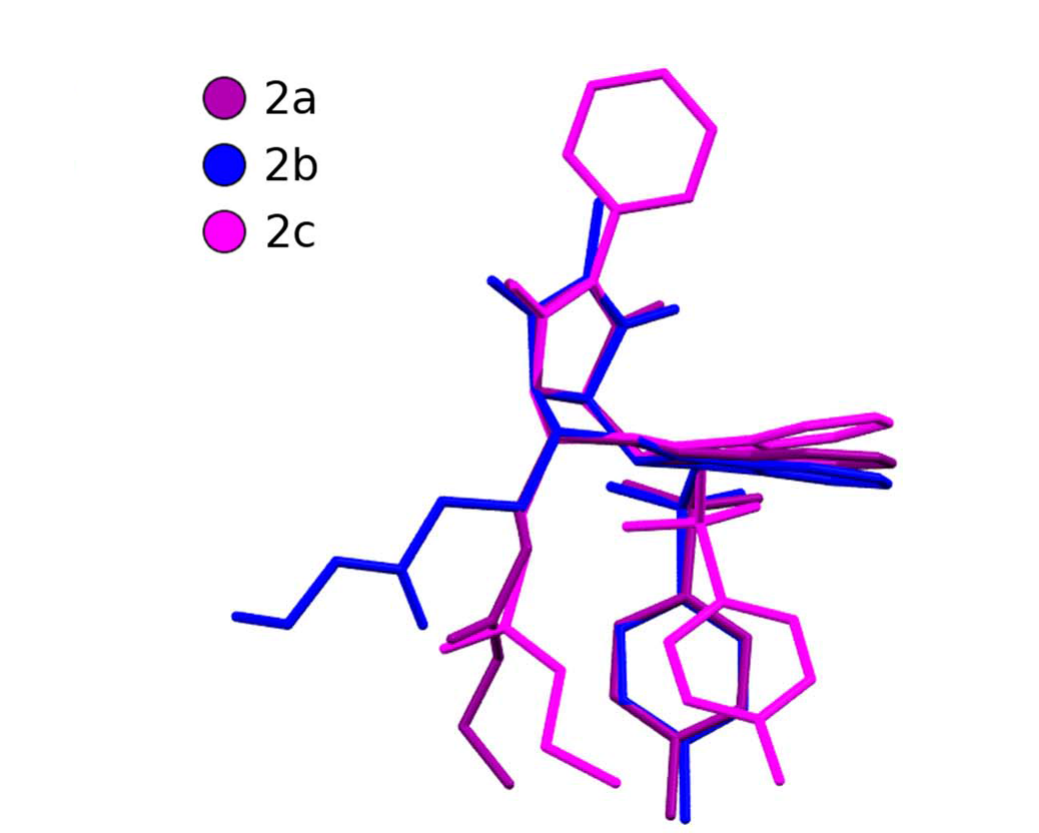
A molecular overlay diagram of the 2,3,9,9*a*-tetra­hydro-1*H*-carbazole molecules of structures **2a**–**2c**. For clarity, hydrogen atoms and water molecules have been omitted and only the disorder components with the highest occupancy are shown. Only molecule A from the asymmetric unit of **2b** has been used in this comparison as all six molecules of **2b** exhibit a relatively similar conformation (see Fig. 5 ▸).

The ability of these molecules to participate in hydrogen bonding appears to correlate to the orientation of the ethyl ester group in the structure (Fig. 6 ▸). Upon further inspection, the molecules of **2a** and **2c** exhibit a quite different conformation to that of **2b**. The orientation of the ester group appears to be key as in both **2a** and **2c** the position of the carbonyl oxygen of this group, directed as it is away from the tosyl group, allows it to act as a hydrogen bond acceptor whereas the same is not possible in **2b** as it is directed towards it.

## 5. Related literature

The following reference is only cited in the supporting information: Betteridge *et al.* (2003 ▸).

## 6. Conclusions

The structure of **2b** is a very interesting case study of a structure in which *Z*′ is greater than 1 can be generated by simple substitution of a single group within a molecular series. The *Z*′ value of 6 is result of a combination of approximate rotation symmetry and a translational modulation distorting a basic *Fdd*2 unit cell into the *Ia* cell observed in the structure. The structure exhibits many of the features commonly associated with high-*Z*′ structures though the lack of strong intermolecular interactions in **2b** is somewhat unusual. Comparing this to two similar structures shows that the lack of conventional hydrogen bonding results in the formation of the dimer units that comprise its unusual asymmetric unit and that this is related to a conformational perturbation of the ethyl ester group common to all three molecules.

## Supplementary Material

Crystal structure: contains datablock(s) 2b, 2c, fdd2. DOI: 10.1107/S2052520626004506/dk5149sup1.cif

Structure factors: contains datablock(s) 2b. DOI: 10.1107/S2052520626004506/dk51492bsup2.hkl

Structure factors: contains datablock(s) 2c. DOI: 10.1107/S2052520626004506/dk51492csup3.hkl

Structure factors: contains datablock(s) fdd2. DOI: 10.1107/S2052520626004506/dk5149fdd2sup4.hkl

Additional information about the analysis of the approximate symmetry in the structure of 2b. DOI: 10.1107/S2052520626004506/dk5149sup5.pdf

CCDC references: 2043589, 2043590, 2524699
