# Supplementary material for: Structural modification of functionalized carbazoles: from Z′ = 1 to Z′ = 6
Source: Acta Crystallogr B Struct Sci Cryst Eng Mater. 2026 May 19;82(Pt 3):339–43. doi: 10.1107/S2052520626004506 (PMC13238489; doi:10.1107/S2052520626004506)
Supplement: Supplementary file 5 [file b-82-00339-sup5.pdf]

Supplementary description of the structure of **2b**
determined at 150 K in space group *Ia* with *Z'* = 6

Analysis of the Structure

The conformations of the six independent molecules are very similar. The *rmsds* for pairwise overlays calculated by *Mercury* (Macrae *et al.*, 2020) are in the range 0.06 – 0.24 Å (average 0.14 Å, *rms* 0.15 Å).

An approximate twofold rotation axis along $[10\bar{1}]_{Ia}$ is very obvious. It relates molecules A&B, C&D, and E&F.

An approximate threefold translation along $[10\bar{1}]_{Ia}$ (or $[\bar{1}01]_{Ia}$) is very obvious. It relates molecules #A,C&E as well as #B,D&F.

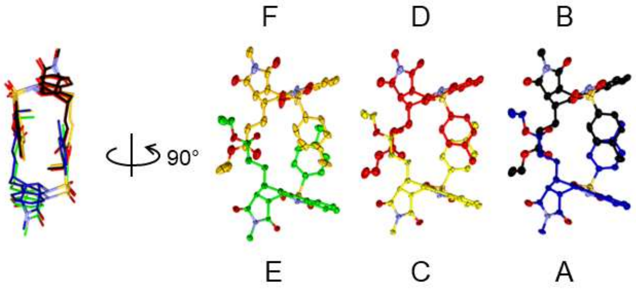

The asymmetric unit showing the approximate twofold axis and the approximate translation. In the view on the left the direction $[10\bar{1}]$ is perpendicular to the drawing; in the view on the right that direction is horizontal. Atomic displacement ellipsoids are shown in the view on the right at the 50% probability level.

The normals of the *a* and *c* glide mirrors of the *Ia* cell are perpendicular to the approximate twofold axis, which is nearly parallel to $[10\bar{1}]$. If the approximate twofold axis and the approximate translations can combine with the glides of *Ia* to give a smaller basic cell in an approximate higher symmetry space group, then the point symmetry of that basic cell must be *mm*2. The point symmetry cannot be *mmm* because the structure is obviously polar, and cannot be of higher symmetry because it is clear that there is no approximate three- or fourfold axis.

If the point symmetry of the basic cell is *mm*2 then there must be an approximate mirror or glide plane having its normal perpendicular to both $[10\bar{1}]$ and **b**. The plane must therefore have indices (*h*0*l*) such that $(h0l)\cdot[10\bar{1}] = 0$. It seemed likely that the plane is (101).

The direction [*uvw*] parallel to the normal of (101) was found visually by looking at a layer (010) viewed along **b** with $[10\bar{1}]$ vertical. The limits for *y* were set at 0.0 – 0.3 because *k* for the plane must be zero and because that range includes the approximate twofold axis $[10\bar{1}]$ at *y* = 0.125. That layer also shows all six molecules without any important overlap. The limits for *x* and *z* were chosen as 0 – 6 and 0 – 2 so that the crystallographic translation approximately perpendicular to $[10\bar{1}]$ would be obvious.

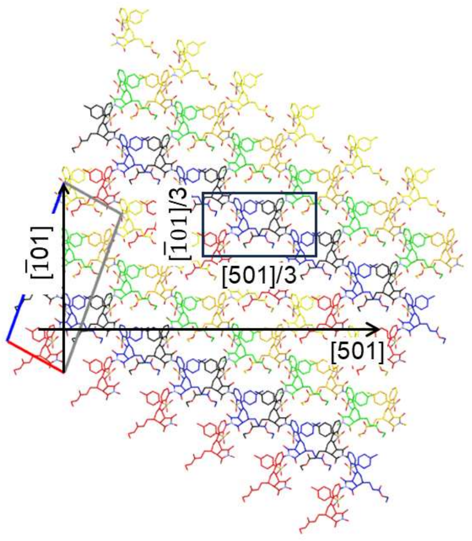

View of a layer (010) showing molecules that have centroids within limits for *x*, *y*, and *z* of 0 – 6, 0.0 – 0.3, and 0 – 2. In this and the following drawings the axes **a**, **b**, and **c** of the *Ia* cell are colored red, green, and blue.)

The translation perpendicular to **b** and approximately perpendicular to (101) is ±[501]. That direction includes an approximate threefold translation because

$[501]_{Ia}/3 = [600]_{Ia}/3 - [10\bar{1}]_{Ia}/3 = [200]_{Ia} - [10\bar{1}]_{Ia}/3$ (or $= [200]_{Ia} + [\bar{1}01]_{Ia}/3$)

The angle between [501] and $[10\bar{1}]$ differs from 90° by 0.017°. The axes of the basic *mm*2 cell then seemed likely to be

$[501]_{Ia}/3$, $\mathbf{b}_{Ia}$, and $[\bar{1}01]_{Ia}/3$,

where the third axis was chosen as $[\bar{1}01]/3$ rather than $[10\bar{1}]/3$ so that the axial system would be right-handed and the direction of **b** would be the same as in the *Ia* cell. (The approximate twofold axis was chosen as **c** rather than as **a** or **b** because the conventional setting of the approximate point group is *mm*2). The cell dimensions are then 22.405, 35.137, and 12.473 Å, and 90, 89.98, and 90°.

The determinant of the transformation matrix

$(5/3\ 0\ ⅓\ |\ 0\ 1\ 0\ |\ \bar{⅓}\ 0\ ⅓)$

is ⅔ so that $Z_{\text{basic}} = (⅔)(6)(4) = 16$. The only *mm*2 space groups that have Z=16 for $Z' = 1$ are *Fmm*2 and *Fdd*2. There is no approximate mirror in this structure so it seemed that the basic cell must have symmetry *Fdd*2. It needed to be shown, however, that the basic cell has *F*-centering and *d* glides.

If the transformation matrix for the cell axes is

$\boldsymbol{a}_{Fdd2} = (5/3\ 0\ ⅓\ |\ 0\ 1\ 0\ |\ \bar{⅓}\ 0\ ⅓)\ \boldsymbol{a}_{Ia}$, where the $\boldsymbol{a}$ are column vectors,

then the transformation for the coordinates is

$\boldsymbol{x}_{Fdd2} = \boldsymbol{x}_{Ia}\ (½\ 0\ \bar{½}\ |\ 0\ 1\ 0\ |\ ½\ 0\ 5/2) + (\Delta x_{origin}\ \Delta y_{origin}\ 0)$, where the $\boldsymbol{x}$ are row vectors.

The two 3x3 matrices are inverses; the additional $(\Delta x_{origin}\ \Delta y_{origin}\ 0)$ vector accounts for the origin shift between the two space groups. The shift along **b** is either +⅛ or -⅛ because the glide planes are at $y = 0$ and ½ in *Ia*, but at $y = (2n+1)(⅛)$ in *Fdd*2. The shift along **a** will be described below. No shift is necessary along **c** because it is a polar direction in both *Ia* and *Fdd*2.

After transformation the centering vector $[½½½]_{Ia}$ becomes $[½½1]_{Fdd2}$, which shows that the new cell is *C*-centered. {Note that the vector $[½½½]_{Ia}$ is the difference of the coordinates *x*, *y*, *z*

and x+½, y+½, z+½ so that the shifts (Δx_{origin} Δy_{origin} 0) cancel.}
The layer $(010)_{Ia}$ shown above suggests that the new cell is B-centered. It is centered because $[\frac{1}{2}0\bar{\frac{1}{2}}]_{Fdd2} = [100]_{Ia}$, which is exact centering, and
$[\frac{1}{2}0\frac{1}{2}]_{Fdd2} = [201]_{Ia}/3 = [100]_{Ia} - [10\bar{1}]_{Ia}/3$, which indicates approximate centering.
If the new cell is both B- and C-centered, then it must be F-centered.

The remaining question is whether approximate d glides are present. In Ia the a glide at y = 0 changes the general position x, y, z to x+½, $\bar{y}$, z. If those two sets of Ia coordinates are taken through the transformation then

<u>coordinates in *Ia*</u>	<u>coordinates in *Fdd*2</u>
x, y, z becomes	$(x+z)/2+\Delta x_{origin}$, $y+\Delta y_{origin}$, $(\bar{x}-\frac{1}{2}+5z)/2$ and
$x+\frac{1}{2}, \bar{y}, z$ becomes	$(x+\frac{1}{2}+z)/2+\Delta x_{origin}$, $\bar{y}+\Delta y_{origin}$, $(\bar{x}-\frac{1}{2}+5z)/2$
	$= (x+z)/2+\Delta x_{origin}+\frac{1}{4}$, $\bar{y}+\Delta y_{origin}$, $(\bar{x}+5z)/2-\frac{1}{4}$

If the first of the $Fdd2$ positions is x', y', z' then the second is $x'+\frac{1}{4}$, $\overline{y'}+2(\pm\frac{1}{8})$, $z'-\frac{1}{4}$, which corresponds to a d glide $[10\bar{1}]_{Fdd2}/4$ with mirror perpendicular to $\mathbf{b}_{Fdd2} = \mathbf{b}_{Ia}$ at y_{Fdd2} = ⅛ (and ⅝) or at -⅛ = ⅞ (and ⅜).
Except for an origin shift, the layer at y_{Ia} = ½ is the same as the layer at y = 0.

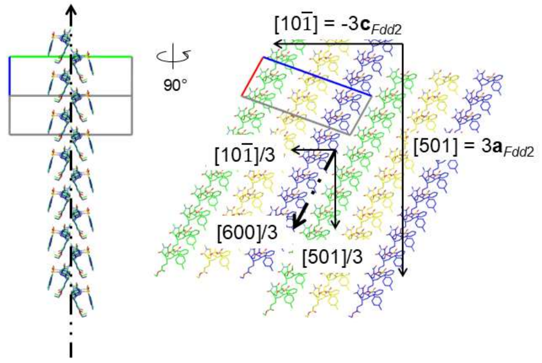

A layer $(010)_{Ia}$ at y_{Ia} = ½ showing the a glide of Ia that becomes a d glide in the approximate $Fdd2$ cell: $[200]_{Ia}$ is $[10\bar{1}]_{Fdd2}$. All directions shown are for the Ia axes unless marked otherwise.

In $Fdd2$ there are two sets of d glides with mirror (010): one set is at y_{Fdd2} = ⅛ and ⅝, and the other is at ⅜ and ⅞; the two sets differ in the sign of the translation along **c** (*i.e.*, [101]/4 or $[10\bar{1}]/4$). Transformation of $[101]_{Fdd2}/4$ gives $[402]_{Ia}/12$, which can be seen to be an approximate d glide in the Ia structure. Transformation of $[10\bar{1}]_{Fdd2}/4$ gives $[100]_{Ia}/2$, which corresponds to the a glide of Ia.

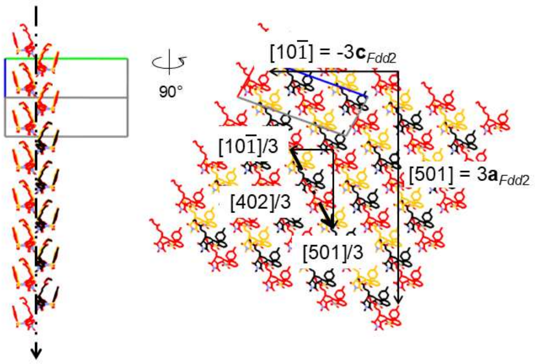

A layer $(010)_{Ia}$ at y_{Ia} = ¼ showing the *c* glide of the *Ia* cell and the approximate *d* glide in the approximate *Fdd*2 cell: $[402]/3_{Ia} = [101]_{Fdd2}$. All directions shown are for the *Ia* axes unless marked otherwise.

While the *c* glide of *Ia* at y_{Ia} = ¼ is present in the approximate *Fdd*2 cell, the *c* glide cannot be described in any simple way because $[001]_{Ia}$ is $[105]/2_{Fdd2}$.

In *Fdd*2 there are also *d* glides with glide mirrors at x_{Fdd2} = ⅛, ⅝ and at ⅜, ⅞. The approximate *d* glides with mirror perpendicular to $\mathbf{a}_{Fdd2}$ (= $[501]/3_{Ia}$) are generated by combining the approximate twofold along $\mathbf{c}_{Fdd2}$ with the glide planes perpendicular to $\mathbf{b}_{Fdd2}$ (= $\mathbf{b}_{Ia}$).

The plane $(100)_{Fdd2}$ is parallel to $(101)_{Ia}$. Transformation of $[011]/4_{Fdd2}$ gives $[\bar{1}31]/12_{Ia}$; transformation of $[01\bar{1}]/4_{Fdd2}$ gives $[13\bar{1}]/12_{Ia}$. Pictures of layers $(101)_{Ia}$ show the approximate *d* glides.

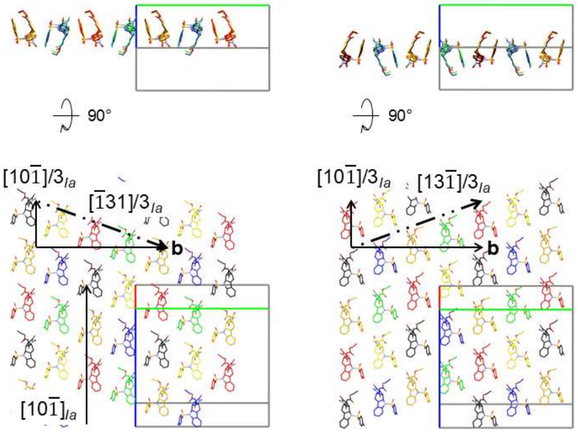

Layers $(101)_{Ia} = (100)_{Fdd2}$ that are separated by Δx_{Fdd2} = ¼ showing the approximate *d* glides with mirrors $(100)_{Fdd2}$. The approximate glide translation $[011]/4_{Fdd2}$ is $[\bar{1}31]/12_{Ia}$; the approximate glide translation $[01\bar{1}]/4_{Fdd2}$ is $[13\bar{1}]/12_{Ia}$.

Analysis of the Twinning

The approximate *Fdd*2 symmetry is so good that it seems almost certain that while being cooled to 150 K the crystal went through a phase transition that did not strongly affect its power to diffract. If so, twinning would be expected, and twinning was found; the *Ia* crystal was a 0.4947/0.5053(7) twin. Such transitions are often called a single-crystal-single-crystal (SCSC) transitions even though twinning necessarily decreases the average domain size.

The twin law was determined by B. Foxman using his program TLAW (https://sites.google.com/brandeis.edu/foxman-group) from the HKLF5 file produced in 2019 or 2020 by the data-processing software. The resulting twin matrix,

$(⅔\ 0\ ⅓\ |\ 0\ \bar{1}\ 0\ |\ 5/3\ 0\ \overline{⅔})$,

corresponds to the individuals of the twin being related by a 180° rotation around $[501]_{Ia}$, which is parallel to $\mathbf{a}_{Fdd2}$.

In the absence of significant resonant scattering, twinning observed in a diffraction pattern can usually be described by any one of the operations of the Laue class of the parent crystal (*mmm* for an *Fdd*2 crystal) that are not preserved in the Laue class of the twinned crystal (2/*m* for an *Ia* crystal). For an *Fdd*2/*Ia* pair in which the direction of the **b** axis is preserved, the possible twinning operations are then reflections with mirrors $(100)_{Fdd2}$ and $(001)_{Fdd2}$ and twofold rotations around $\mathbf{a}_{Fdd2}$ and $\mathbf{c}_{Fdd2}$. In the crystal of this study, plane $(100)_{Fdd2}$ is parallel to $(101)_{Ia}$ and $(001)_{Fdd2}$ is parallel to $(\bar{1}05)_{Ia}$. Rotation axis $\mathbf{a}_{Fdd2}$ is parallel to $[501]_{Ia}$ and $\mathbf{c}_{Fdd2}$ is parallel to$[\bar{1}01]_{Ia}$. Twinning by reflection across $(001)_{Fdd2}$ or by a 180° rotation around $\mathbf{a}_{Fdd2}$, however, is very unlikely in an SCSC transition from an untwinned parent crystal because both those operations would reverse the direction of the very polar axis $\mathbf{c}_{Fdd2}$ (parallel to $[\bar{1}01]_{Ia}$). The twinning seems therefore to have been by reflection across $(100)_{Fdd2}$ [parallel to $(101)_{Ia}$] and/or by rotation around $\mathbf{c}_{Fdd2}$ (parallel to $[\bar{1}01]_{Ia}$). This study cannot distinguish between those two possibilities.

The twin matrix given above is for a rotation because standard crystallographic software always chooses the rotation so that the results can be correct for an enantiomerically pure crystal. The matrix corresponds to a rotation around $[501]_{Ia}$ (parallel to $\mathbf{a}_{Fdd2}$) rather than around the more likely $[\bar{1}01]_{Ia}$ (parallel to $\mathbf{c}_{Fdd2}$) as a result of the indices assigned to the twin components by the processing software, which has no information about the structure. All the directions given above may, of course, be inverted.

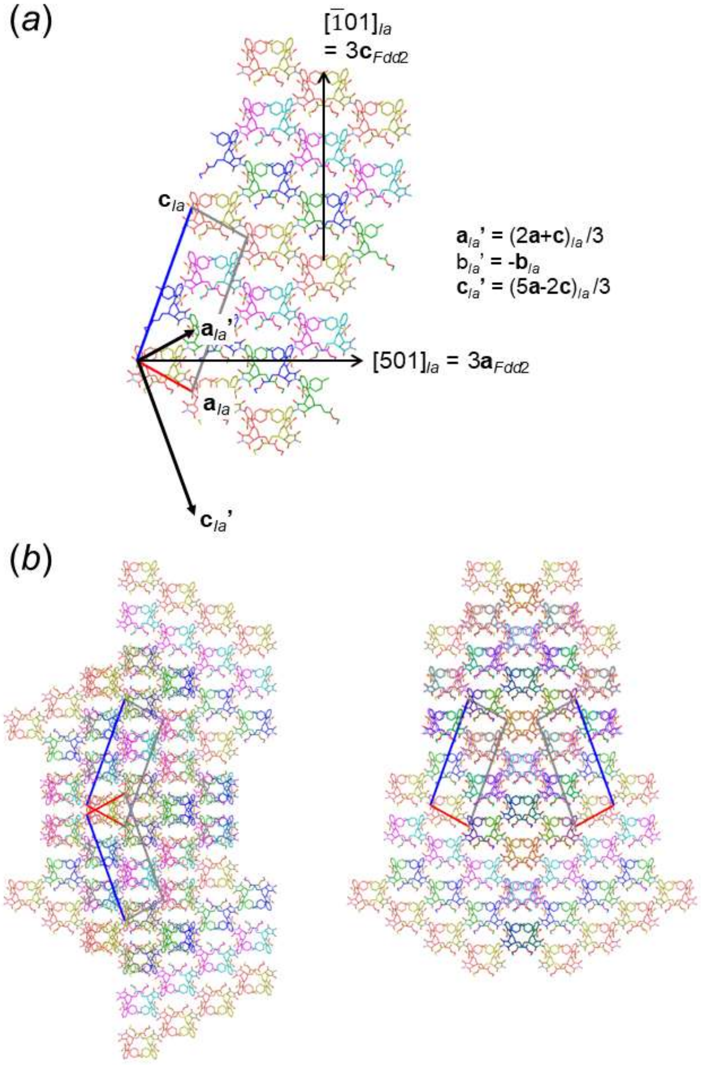

Illustration of the twinning. (a) Diagram showing the relationship of the axes of the two components of the twin as determined by the program TLAW. The axes $\mathbf{a}_{Ia}$**'** and $\mathbf{c}_{Ia}$**'** are those of the second component. (b) Overlay with translation of the two components as related by (left image) the rotation around $[501]_{Ia}$ or reflection across $(\bar{1}05)_{Ia}$, and (right image) by rotation around $[10\bar{1}]_{Ia}$ or reflection across $(101)_{Ia}$. The twin operations for the image on the left invert the very polar direction $[10\bar{1}]_{Ia}$ but the operations for the image on the right do not.

To distinguish between twinning by rotation or by reflection B. Foxman carried out a four-component refinement by adding two additional components to the original HKLF5 file, with the four components being the parent structure and the three structures related to it by inversion, twofold rotation, and reflection through a mirror. The reflection file was imported and checked using the TLAW option in the *Crystals for Windows* program (Betteridge *et al.*, 2003). This refinement was inconclusive; the uncertainties were large enough that it was impossible to determine whether the twinning is better described by a twofold rotation or a mirror operation, probably because there is little resonant scattering in this $C_{27}H_{28}N_2O_6S$ structure. The Flack parameter given in the CIF from the original refinement is 0.46(4).

It is also possible that the presumed *Fdd*2 parent crystal was itself a twin in which the individuals had axes $\mathbf{c}_{Fdd2}$ pointing in opposite directions.

While it would be desirable to determine this structure at room temperature, where the space group would be expected to be *Fdd*2, doing so is not feasible. The original crystals are no longer available (the cif was created in January 2020), and synthesizing them again would require more work than can be justified.

Refinement of the Basic Structure in *Fdd*2

If the *Ia*, *Z'*=6 structure is the result of a phase transition from an *Fdd*2, *Z'* = 1 structure, then it should be possible to refine the average *Fdd*2 structure using the *Ia* reflections that would have integral indices when transformed to the *Fdd*2 basic cell.

Data for the refinement in Fdd2

The HKLF5 file from the *Ia*, *Z'* = 6 refinement was imported into a spreadsheet, where the indices of the 21138 reflections were transformed as

$\boldsymbol{h}_{Fdd2} = (5/3\ 0\ 1/3\ |\ 0\ 1\ 0\ |\ \overline{1/3}\ 0\ 1/3)\ \boldsymbol{h}_{Ia}$, where the $\boldsymbol{h}$ are column vectors.

In *Fdd*2 the contributors to the 5968 overlapped reflections have indices *hkl* and $hk\bar{l}$, and so should have the same intensity in the absence of significant resonant scattering. Of those 5968, 5040 have integral indices in *Fdd*2. In the HKLF5 file the *I* and $\sigma(I)$ values are exactly the same for the two contributions to an overlapped reflection; the values and indices for component 1 were retained and those for component 2 were discarded.

There are 4 reflections from component 1 and 51 from component 2 that are not overlapped and that have integral indices in *Fdd*2. All but 3 of the 51 are represented in the list of 5040 overlapped reflections by reflections that would be equivalent under orthorhombic symmetry in the absence of significant resonant scattering. The 3 that are not represented have $I/\sigma(I)$ values of 2.6, 2.5, and 0.6 and so were judged to be unimportant.

The *I* and $\sigma(I)$ values of the 5040 overlapped reflections with integral indices in *Fdd*2 were included in the refinement of the higher-symmetry structure. In the *Shelxl* refinement 69 of the 5040 were deleted as systematically absent, and the number of unique reflections after symmetry averaging (MERG 2) was reported to be 3812. The R_{int} value for the averaging was given as 0.028.

Atomic coordinates for the refinement in Fdd2

A view of the *Ia* structure showed that molecule F, after a translation [$\bar{1}$00], would be a good starting point. The coordinates of shifted molecule F were then transformed as

$\boldsymbol{x}_{Fdd2} = \boldsymbol{x}_{Ia}\ (1/2\ 0\ \overline{1/2}\ |\ 0\ 1\ 0\ |\ 1/2\ 0\ 5/2)$, where the $\boldsymbol{x}$ are row vectors, and the matrix is the inverse of the matrix that relates the cell axes and Miller indices.

The origin of *Ia* is fixed along **b** only; the origin of *Fdd*2 is fixed along both **a** and **b**. The origin shift along **b** is either +⅛ or -⅛ (see above). The origin shift affecting the coordinates *x* still needed to be determined.

The asymmetric unit of the *Ia* structure is a compact set of molecules related by the approximate twofold axis and the approximate translation [10$\bar{1}$]/3. The centroid of that unit in the *Ia* cell is

0.860, 0.625, 0.533 (*i.e.*, 0.860, ⅝, 0.533)

and when transformed to the *Fdd*2 cell is

0.6965, 0.625, 0.9025 (*i.e.*, 0.6965, ⅝, 0.9025)

In *Fdd*2 there are axes 2 when *x* and *y* are both = 2*n*(¼) and when *x* and *y* are both = (2*n*+1)(¼). The necessary shift vector therefore has *y* = +⅛ or = -⅛, and *x* = +0.0535 or = -0.1965.

The shift vector [-0.1965, -⅛, 0] was determined to be the correct choice. The three other possibilities (*i.e.*, [+0.1965, -⅛, 0], [-0.1965, +⅛, 0], [+0.1965, +⅛, 0]) all gave different results, and all led to contacts between molecules so short that they were marked by *Mercury* as covalent bonds.

Results of the refinement in Fdd2

A standard refinement with *Shelxl* was performed (non-H atoms anisotropic; H atoms in calculated positions). The final R_1 [$I/\sigma(I) > 2$] and wR_2 values were 0.069 and 0.196. While the atomic ellipsoids are not completely consistent with expected low-frequency modes, the ellipsoid are not especially suspicious. The refinement in the approximate *Fdd*2 cell would be found acceptable for publication by most journals. The cif for this refinement has been deposited.

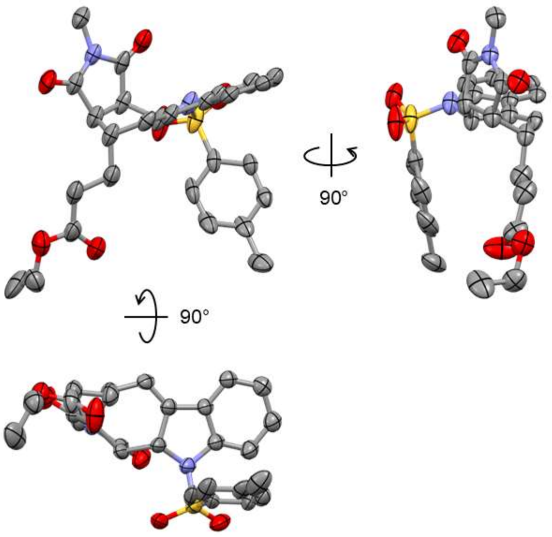

Views of the atomic displacement ellipsoids (50% probability level) from the refinement in *Fdd*2 using the 150 K *Ia* data that have integral indices when transformed to *Fdd*2. The effects of the averaging of the six independent molecules are most obvious in the view at the top left.

Analysis of the Intermolecular Interactions

Pairs of molecules form compact dimers having near perfect twofold rotational symmetry. The dimers include short C-H…O=C contacts that are probably stabilizing. While there is no simple way of proving that these dimers are more favorable than a dimer with approximate inversion symmetry, attempts to maneuver two molecules into a centrosymmetric arrangement suggest that the observed arrangement is more compact. It is possible that the dimers are present in the solutions from which crystals were grown.

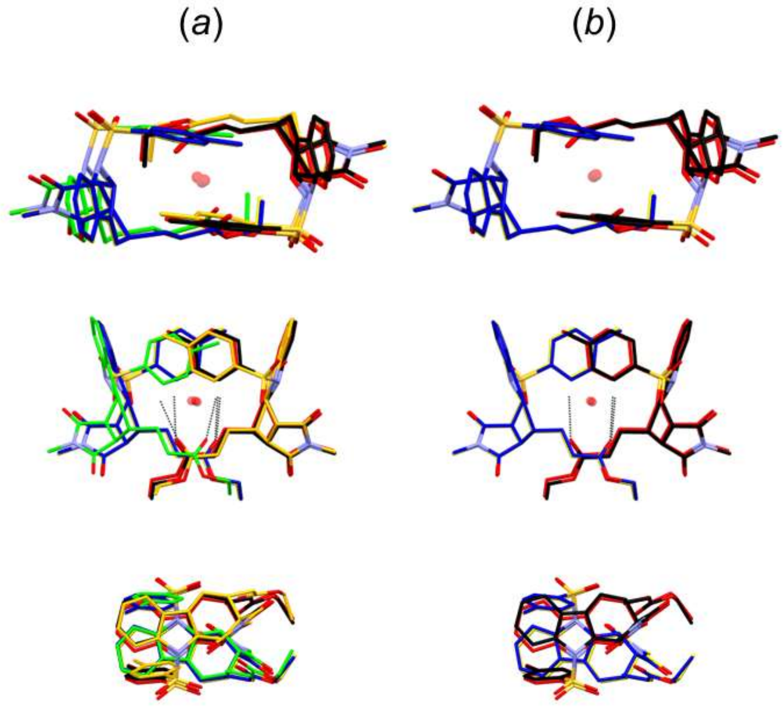

(*a*) Three views of the overlaid dimers. The dimer centroids (calculated without H atoms) and the short CH…O=C contacts are shown. The top view is along $[10\bar{1}]_{Ia} = \mathbf{c}_{Fdd2}$; the other two superpositions were constructed manually. (*b*) The same views but without the dimer of molecules E and F. A comparison of (*a*) and (*b*) shows that the E&F dimer differs some from the other two.

The intermolecular CH…O= contacts shorter than $\Sigma_{VDW} - 0.1$ Å are mostly within layers (010) with $0 < y < ¼$, $¼ < y < ½$, *etc*. that include the approximate twofold axes Those layers are homochiral; adjacent layers are related by glides.

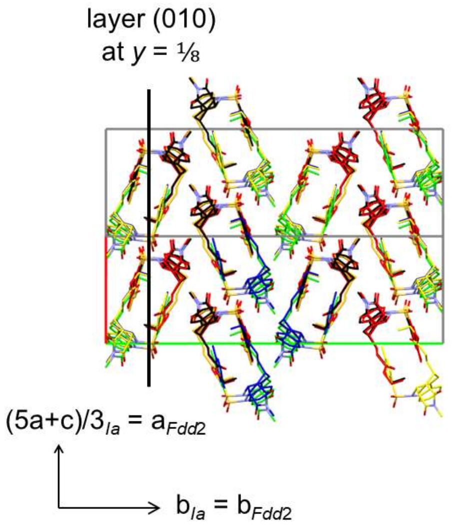

Projection of the structure along $[10\bar{1}]_{Ia} = \mathbf{c}_{Fdd2}$. A layer (010) at $y = ⅛$ is marked.

Within the layers the important contacts are between dimers related by $[100]_{Ia} = [10\bar{1}]_{Fdd2}/2$ or by $[201]_{Ia}/3 = [101]_{Fdd2}/2$, *i.e.*, between dimers related by the approximate B_{Fdd2} centering. These contacts seem to favor all dimers being oriented in the same direction, so that the layer has a polar axis.

The CH…O= contacts between layers (010) are between molecules related by *d* glides so that the whole structure is polar.

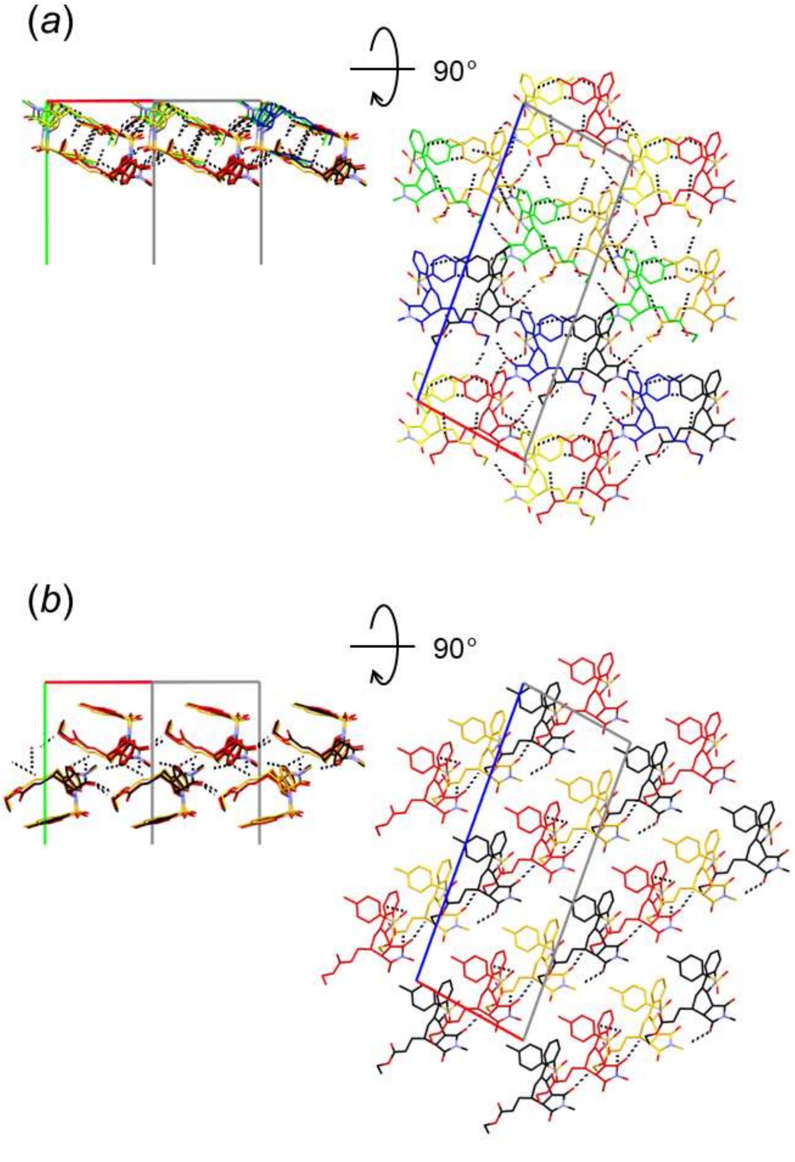

(*a*) Projections of a layer (010) at $y = \frac{1}{8}$ showing the intermolecular contacts shorter than $\Sigma_{\mathrm{VDW}} - 0.1$ Å that are within the layer. (*b*) Projections of a layer (010) at $y = \frac{1}{4}$ showing the intermolecular contacts shorter than $\Sigma_{\mathrm{VDW}} - 0.1$ Å that are between layers. In both (*a*) and (*b*) the direction $[10\bar{1}]_{Ia} = \mathbf{c}_{Fdd2}$ is vertical in the image on the right.

Acknowledgment

We are very grateful to Emeritus Professor Bruce M. Foxman of Brandeis University for his help with analyzing the twinning.

References

Betteridge, P. W., Carruthers, J. R., Cooper, R. I., Prout, K. & Watkin, D. J. (2003). *J. Appl. Cryst*. **36**, 1487.

Macrae, C. F., Sovago, I., Cottrell, S. J., Galek, P. T. A., McCabe, P., Pidcock, E., Platings, M., Shields, G. P., Stevens, J. S., Towler, M. & Wood, P. A. (2020). *J. Appl. Cryst*. **53**, 226-235.
